# Population Health Management Approach: Integration of Community-Based Pharmacists into Integrated Care Systems: Reflections from the U.S., Achievements in Scotland and Discussions in Germany

**DOI:** 10.5334/ijic.5431

**Published:** 2020-06-25

**Authors:** Lauren F. Lyles, Helmut Hildebrandt, Alpana Mair

**Affiliations:** 1 Alexander von Humboldt Foundation, DE; 2 OptiMedisAG, DE; 3 The Scottish Government, GB

**Keywords:** Community Pharmacists, Collaborative Practice Agreements, Prescription for Excellence, Population Health Management, Gesundes Kinzigtal, Polypharmacy Management

## Abstract

The annual amount spent on healthcare per capita is higher and expected to grow in the U.S. compared to healthier level 4 countries (e.g., United Kingdom, Canada, Germany, Australia, Japan, Sweden, Netherlands), while health outcomes continue to be suboptimal [1,2,3]. Therefore, healthcare is slowly shifting from a fee-for-service to value-based care, which addresses social determinants of health, promotes outcome-based contracting and employs more Population Health Management (PHM) activities. The root cause for this shift has been the increase in patients’ out-of-pocket costs and the pervasiveness of poorer outcomes. PHM has been defined by many as a mindset and activities that support the Triple Aim Initiative (i.e., **improving population health, experience of care, reducing costs**) [4].

This article outlines the value of pharmacists on health outcomes in the U.S., Germany, and Scotland and innovative PHM approaches through pharmacist collaborative networks, polypharmacy management and pharmacists’ integration in care models [1,5].

## Reflections from the U.S.

### Pharmacists Positioned for Population Health Management

The pharmacy profession has always strived to overcome its traditional stereotype solely as a ‘medication dispenser’, to become recognized as the ‘medication expert’. Through advocacy, education (Bachelors to Doctor of Pharmacy, Pharm. D.), and policy advances, pharmacists have become more integral members of the healthcare team in the U.S., and one of the most trusted professionals along with nurses and physicians, according to Gallup [7].

The U.S. Department of Health and Human Services Center for Medicare and Medicaid has also supported this argument by advocating for expanded pharmacists’ services (e.g., offering tobacco cessation by initiating nicotine replacement therapy, managing opioid overdose by dispensing Naloxone, immunizing for influenza viral infection) by way of prescriptive authority, collaborative practice agreements (CPA) with physicians, standing orders by the state, or other prearranged protocols and partnerships [8]. Therefore, majority of states have re-engineered their ‘policies’ and ‘thinking’ to support pharmacist-patient centered care services to address gaps in care and expand patient access [8,9].

The role of a pharmacist may now include initiating clinical and non-clinical interventions in disease-state management programs, rationalizing cost-effective use of evidence-based medicines, promoting healthy lifestyles through wellness screenings, providing immunizations, medication therapy management and patient counseling [10,11].

The advancements of pharmacy practice in the U.S. have subsequently positioned pharmacists for population health management, especially in the community setting [12].

### Population Health Management: A Sustainable Solution

A sustainable solution for better management of healthcare dollars and enhancing patient outcomes is the application of population health management strategies and activities [13]. The multifaceted approaches of PHM include the following strategies: (Table 1).

**Table 1:**
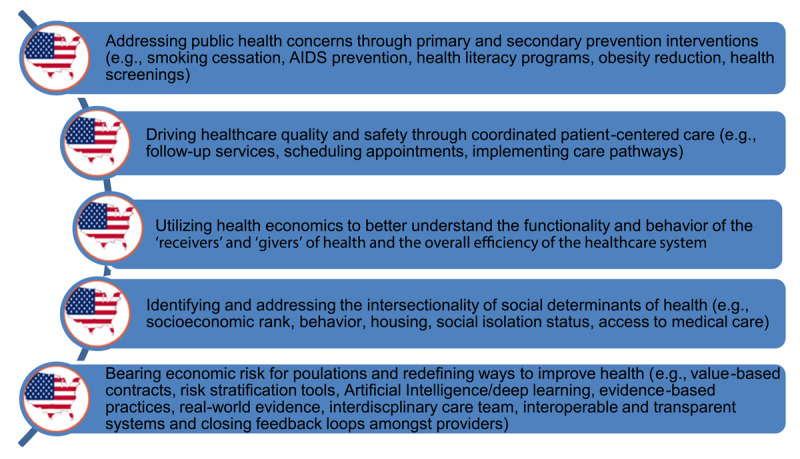
Elements of Population Health Management.

### Everyone Has a Role

In the U.S., the pharmacy sector is filled primarily by corporate chains, which operate over 40,000 community pharmacies followed by an estimated 21,909 small business community pharmacies or ‘mom and pop’ pharmacies with roughly 1,800 rural independent pharmacies serving as the only pharmacy provider in their community [14,15].

With an overarching presence from urban to rural zip codes, community-based pharmacists are able to support patient care by building trust and collaborating with other healthcare professionals. Despite the chronic tensions between scopes of practice, it is true that ‘Everyone has a Role’ in the future of population health management. However, it is unequivocally important to also look at the data regarding physician shortages and the number of chronic patient visits to their pharmacy averaging 35 times annually compared to seeing their providers an average of 3.5 per year. This allows for unique opportunities for pharmacists to help meet the Triple or Quadruple Aim in a value-based care setting [6,16,17,18]. Literature also reflects that pharmacist-provided services help mitigate risk of adverse drug events, medication errors, improve patient outcomes and are cost-effective [19].

### Evidence of Community Pharmacists Enhancing Care

Community pharmacists are one of the most accessible and qualified healthcare professionals in the U.S., Scotland, and in Germany to provide patient-centered care. According to the Institute of Medicine, medication errors harm approximately 1.5 million patients in the U.S. annually, and generates over 3 billion dollars in medical costs [20]. The data may suggest there is an opportunity to better utilize and integrate pharmacists’ accessibility and expertise to aid multidisciplinary teams and patients in the mitigation of medication errors and medical costs.

Since the 1970s, community pharmacists have developed collaborative practice agreements (CPAs) as their integrative avenue to provide and receive reimbursement for patient care services and coordinate care with physicians. CPAs create a formal agreement that varies state by state between the licensed physician and pharmacists; the physician authorizes the CPA under protocol-specific patient care functions that outline what services the pharmacists are able to provide [11].

To illustrate, pharmacists in Ohio under a CPA may contract with multiple providers in order to manage the patient’s full drug profile, order laboratory test and modify drugs based on the patients’ results [17]. Other pharmacist-patient care services, include Medication Therapy Management (MTM) and Collaborative Drug Therapy Management (CDTM), which reduce care fragmentation as well as optimize health outcomes and lowering costs [18].

The Chisholm-Burns’ study of 2010 also showed that patients improved significantly when pharmacists worked in tandem with physicians and others in managing the entire patient. The meta-analysis brought to life the pharmacist’s engagement in interdisciplinary health management, which dramatically improved patients’ blood pressure, hemoglobin A1C, lowered cholesterol levels, and reduced adverse drug events. Pharmacists’ patient care services also mitigated division in care, cut health expenditures, and enhanced health outcomes [21].

One of the most touted studies in pharmacy-patient history is the Asheville Project along with the Patient Self-Management Program for Diabetes and the Diabetes Ten City Challenge [22,23,24,25]. These were the efforts by self-insured employers, to better manage the health of their employee population with chronic diseases such as diabetes, hypertension and high cholesterol [25]. The patient populations were enrolled in collaborative care programs with a community pharmacist on each care team. The outcomes were substantial savings, improved population health and improved prevention measures [22,23,24,25]. Despite the aforementioned articles underlining the value of pharmacists in population health, there are still weaknesses and threats (e.g., reimbursement challenges, outdated statutes, competency knowledge gaps between healthcare professions, limited access to Electronic Health Records (EHRs) and other health technologies) that create barriers for pharmacists to smoothly integrate in holistic and coordinated patient care.

### Opportunities

Organizations such as the *Community Pharmacy Enhanced Services Network* (CPESN® USA) have created a clinically integrated network of pharmacies with 2000+ pharmacies, as of March 2019. CPESN USA focuses its efforts on showing the value and outcomes of community-based pharmacists among providers and populations by applying population health activities through their enhanced pharmacy services within their local community-based pharmacies. Enhanced pharmacy services (e.g., medication synchronization, reconciliation, immunizations, medication reviews) are required for each participating CPESN community-based pharmacy, and some are encouraged to offer additional innovative services such as opioid patient support and provider education, HIV patient support, and disease state management and education programs [26].

With the culture shift of paying for outcomes to show value, CPESN USA developed the Pharmacist eCare Plan as a platform for recording pharmacist-patient assessments (patient goals, medication list, and medication-related problems). The eCare Plan is an opportunity for mutual health information exchange between different pharmacies [27]. The future state of the Pharmacist eCare Plan is to go beyond recording and actually leveraging its interoperable nature for communicating pharmacist interventions to other networks, community-based pharmacies and partners (Accountable Care Organizations, primary care clinics, hospitals and more). It also allows for pharmacists to showcase accountable and transparent care for their community, initiate proper reimbursement models with payers, and support coordinated-care efforts.

A recent study including community pharmacies and community health centers in integrated care programs providing comprehensive medication management, has also demonstrated how logistics such as documentation burden can be the rate limiting step for pharmacists integration [28]. Pharmacists in successful integrated care settings have shown favorable impacts on multiple aspects of patient care, including pharmacist-managed population health management programs by improving blood pressure in patients with coronary artery disease, improved inappropriate prescribing patterns, and decreased drug interactions [29,30]. With implementation of new value-based care delivery models and population health management, pharmacists and organizations such as CPESN USA will integrate their expertise in the larger healthcare paradigm.

## Achievements in Scotland

### From Pharmacist to Pharmacist-Prescriber: Ensuring Appropriate Polypharmacy Management

In the U.K., the healthcare provision is devolved to the four nations so that each has their own services for delivering care that draws on the expertise of pharmacists. This section will focus on some of the developments particularly in relation to appropriate polypharmacy management and medication management by pharmacists that has been developed in recent years.

With an aging population and the associated increase in multiple morbidities, more people are taking multiple medications (polypharmacy). The associated polypharmacy may be appropriate or inappropriate, but as most clinical guidance is based on a single disease, often polypharmacy becomes inappropriate when the risks outweigh the benefits from the medication. With increasing age there is the potential for renal and hepatic impairment, hence decreased clearance of drugs, and increased susceptibility to side effects. Polypharmacy is a public health challenge and a source of unnecessary harm and unnecessary spending in some cases. The IMS report stated that 0.3% of the total health expenditure, which is $18bn could be avoided if inappropriate polypharmacy was managed adequately [31].

**Figure 1:**
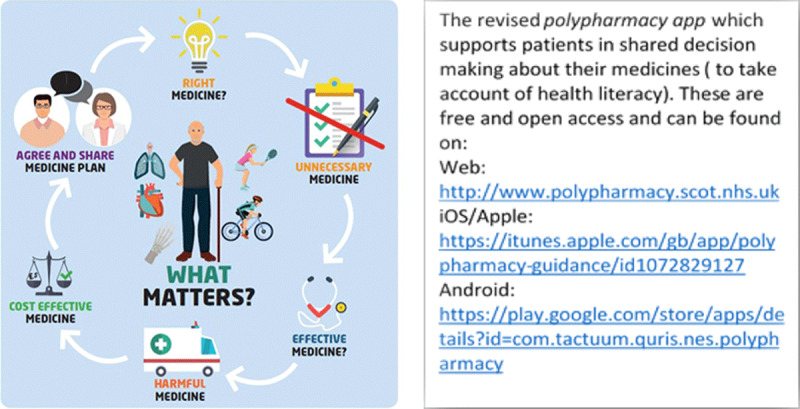
7 Steps to appropriate polypharmacy.

It is estimated that worldwide, 3–6% of all hospital admissions are attributed to medicines. In the U.S., the figures range from 2–19% and in the U.K. reporting up to 11% (1,16–18%). If this latter figure was extrapolated across the European Union, there would be 8.6 million unplanned admissions due to medication related side effects with half being preventable. At least 70% of unplanned admissions are in people over the age of 65 taking 5 or more medications. With all these factors in mind, in 2012 the first Scottish Polypharmacy Guidance was published. Throughout Scotland, a directive was issued by the Director General Health and Chief Executive of NHS Scotland directing pharmacists and general practitioners to address the problem of polypharmacy in the elderly, especially the frail through polypharmacy management services or interventions. In order to standardize an appropriate polypharmacy review, a seven step process was developed after several iterations with the key question of “What Matters to You”? (Figure 1) This emphasizes the population health management elements of patient education and coordinated care, which ensure that patients take an active role in their review and are involved in shared-decision making [32].

The program in Scotland was implemented using Kotter’s Change Management and key lessons learned from Scotland’s lead role in the European Union project, SIMPATHY, which addresses polypharmacy in the elderly [33]. These lessons, include: (Table 2).

**Table 2:**
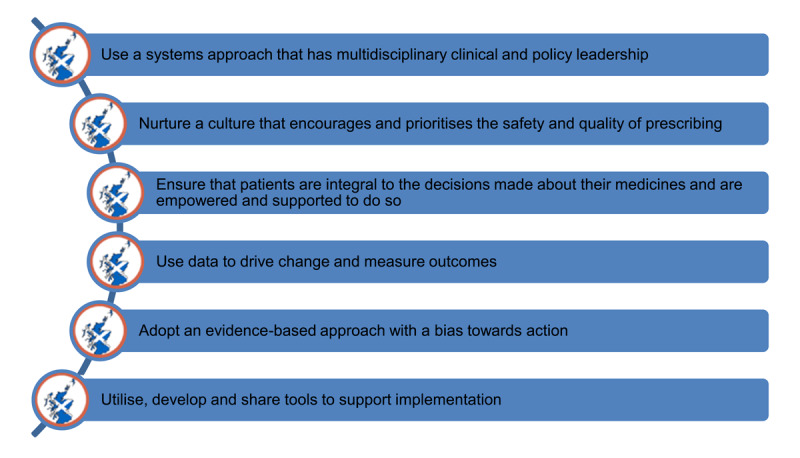
Kotter’s Change Management and Scotland’s Key Lessons Learned from SIMPATHY Polypharmacy Project.

**Figure 2:**
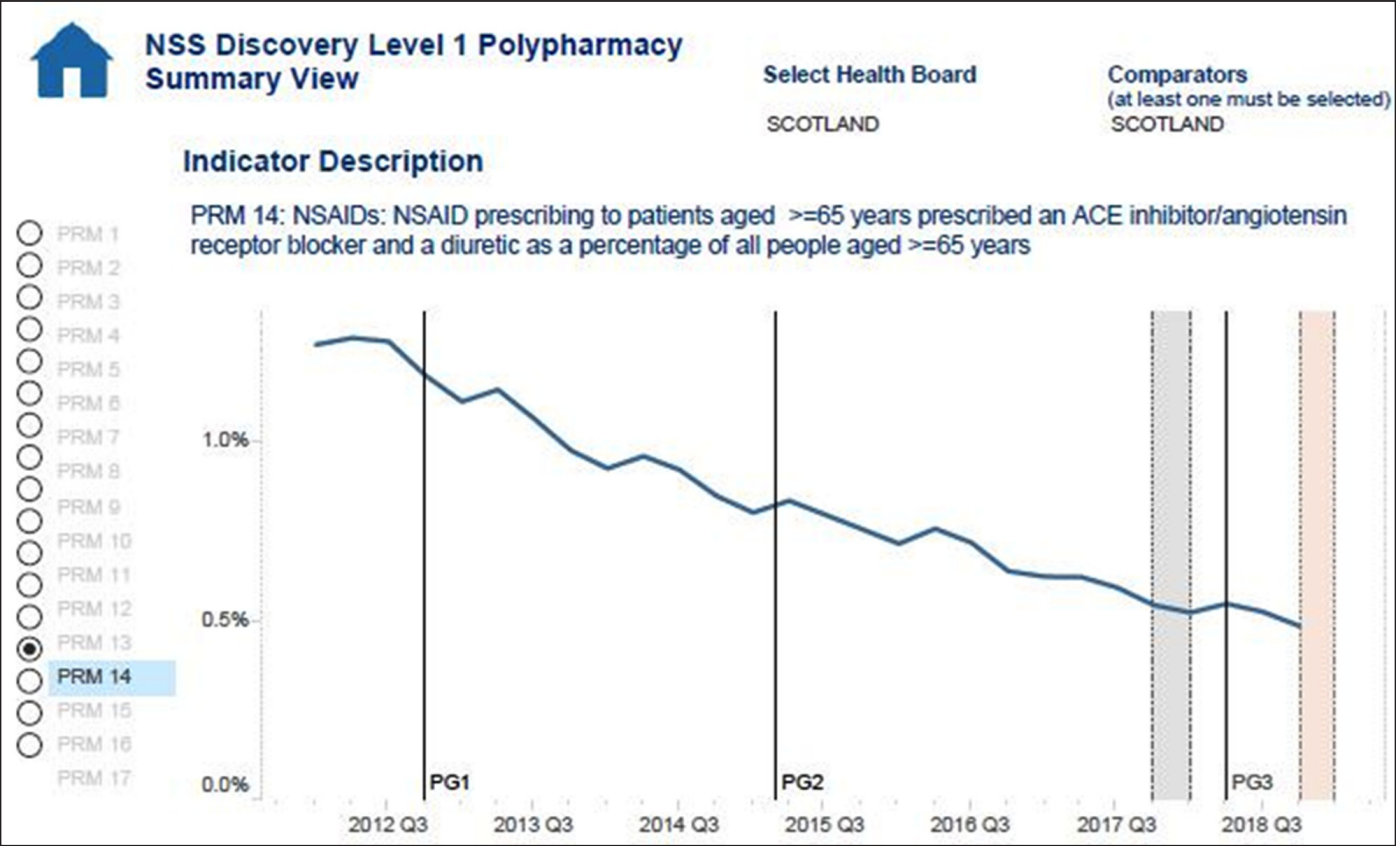
To show national data (Scotland) reductions of high-risk drug combinations (NSAIDs with ACEI/ARBs and diuretics) [34].

In 2013, there was also the introduction of undertaking this work in the general practitioners contract in Scotland. Economic evaluation through each of the guidance has shown that the polypharmacy reviews reduce the number of medications that individuals take by 1–2 medications per person, saving approximately £120 per person per year. In order to record improvements, indicators were developed. One such example is the rate of the ‘triple whammy’ which is the combined use of non-steroidal anti-inflammatory drugs, diuretics and angiotensin converting enzyme inhibitors Figure 2.

In 2013, the Prescription for Excellence action plan was published to promote improved coordinated care between pharmacists, physicians and other healthcare workers across the healthcare system and to support appropriate management of multiple morbidities, in particular medication management. Pharmacists needed to train as independent prescribers in order to implement these services and work in partnership with the general practitioners. Pharmacists were able to take on a case load of patients and manage their polypharmacy. By working as independent prescribers, this creates the autonomy to change and review medications while creating additional capacity in general practice and in hospital settings to review more patients.

**Figure 3:**
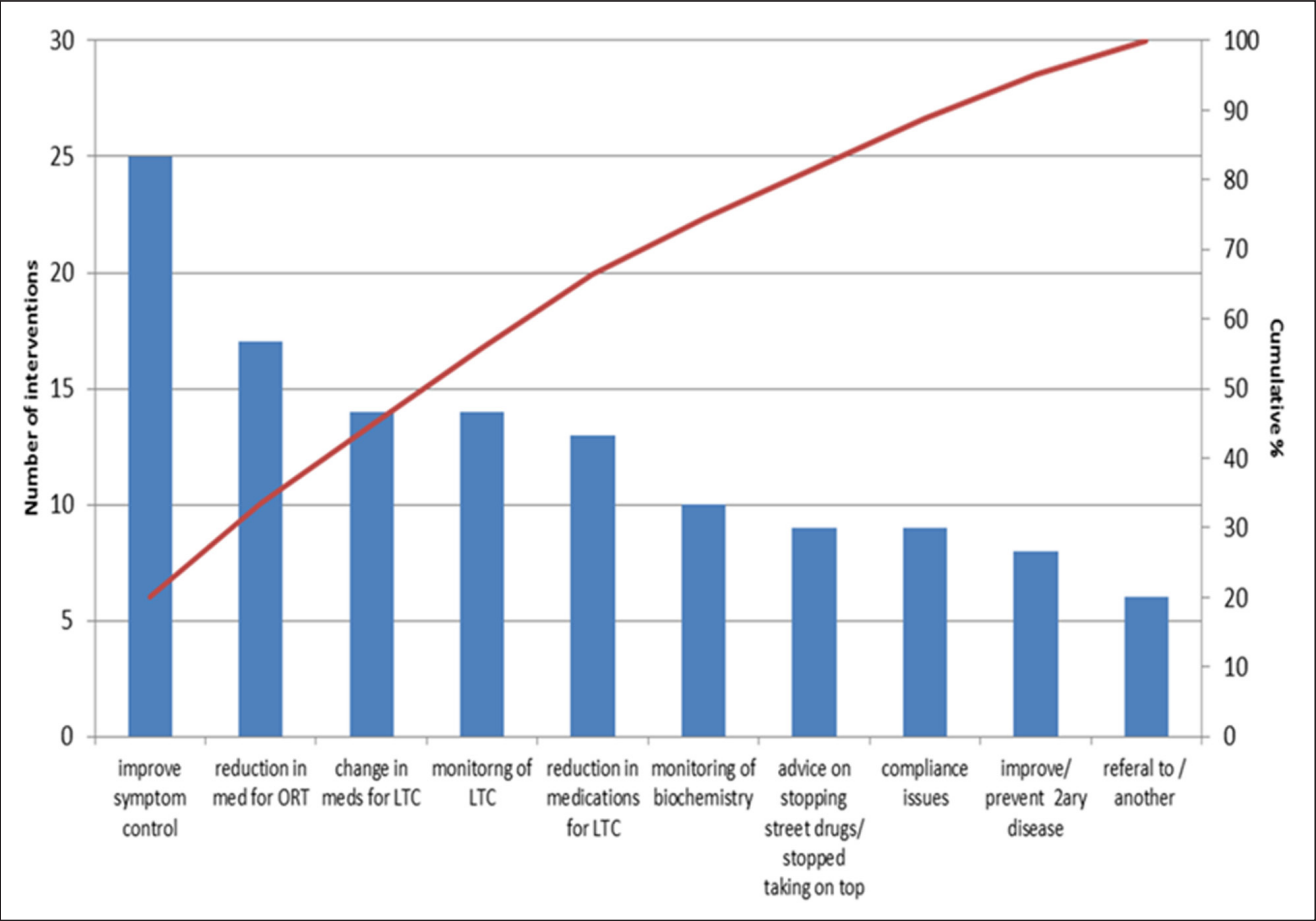
Chart to show that 80% of interventions were for improve managment of LTC (long term conditions) and help with reducing medcines for substance misuse.

With an increasing demand on primary care due to an aging population, and the increasing number of medications taken by patients, the Cabinet Secretary for Health committed to ensuring that pharmacists were integrated into the primary care practice. This would affect all general practitioners practices across Scotland. Prescription for Excellence also highlighted that the improvement in appropriate prescribing should be across health and social care settings. The importance of this is illustrated by pharmacists of primary care practices outreaching to the homeless population and providing clinical interventions to improve the population’s outcomes. For example, in a pilot study of 46 patients in homeless shelters, a number of interventions were pharmacist-led for the homeless population, including: medication management, disease state management, screening patients with COPD and offering pain management services. This work has been successfully implemented and a caseload of patients is managed by pharmacists. (Figure 3).

In Scotland, pharmacists were provided with additional clinical skills training and trained as independent prescribers, meaning they are able to prescribe and change treatments for their patient population. The transformative Prescription for Excellence policy for pharmacists’ prescriptive authority in GP Practices has been adopted across the UK. The role for pharmacists in general practice was open to all pharmacists including some community pharmacists who will go into the GP practices to deliver these services at disease specific clinics (e.g., asthma clinics) It was also recognized that community pharmacists are ideally placed to be the first port of call for patients with common clinical conditions and polypharmacy to alleviate the burden off people going to the general practitioner. These services are currently being developed for implementation in community pharmacies for all people, beginning with the management of simple urinary tract infections, management of self-management plans for COPD and treatment of impetigo [35].

## Discussions within Germany

Population Health Management – PHM is discussed in the academia and the pressure for stronger networking of the different sectors in healthcare has reached the policy arena in Germany and was mentioned in the last coalition agreement of the government in Germany. But so far there are only some initiatives which claim to implement Population Health Management on a regional basis. Some of them have been evaluated and have already been able to deliver their “proof of concept”, such as the integrated care in the “Gesundes Kinzigtal [Healthy Kinzig Valley]” project, supported by OptiMedis, a company oriented towards Population Health Management with a strong scientific focus, and a local network of doctors [36]. The contractual background for the solution is a shared savings model with health insurance companies that have more than 50% of the statutory health insurance (public insurance) policyholders in this region (AOK and SVLFG).

### Pharmacists’ Involvement in a Pilot of Integrated Care “Gesundes Kinzigtal”

Gesundes Kinzigtal GmbH has built a health network of numerous local partners and is able to invest about 3 million € each year into the health of the insured through a variety of health care management services as well as health and prevention programs because of its share of the savings that are produced by these activities [37]. Although the Kinzigtal solution has long been managed by a pharmacist, the practical involvement of pharmacists there has been unfortunately somehow limited.

Participation of pharmacists in the integrated care “Healthy Kinzig Valley” can be summarized as follows:

- Continuous participation of two pharmacist representatives of the local pharmacies and the pharmaceutical department within the regional hospital in a drug commission and discussion of data evaluations of the drug supply for the population concerned (around two meetings per year).
- Smoking cessation program in Kinzigtal by getting paid directly by “Gesundes Kinzigtal” for dispensing Varenicline to program participants
- Research project to provide drug blister packs for approx. 100 chronically ill patients on therapy with more than four drugs.
- Training of several local pharmacists in medication review and subsidization of individual reviews
- Attempt at participation with referral of chronically ill patients to patient and prevention coaching

Many other development solutions in Germany have arisen from alliances and mergers between doctors, who in turn have designed selective contracts with health insurance companies, but with no or relatively little cooperation with pharmacists. Traditionally, cooperation between physicians and pharmacists in Germany has been limited to positive individual cases, whereas at the association level it has been burdened with mutual reservations and demarcations (according to the motto “Schuster bleib bei deinem Leisten”, “Cobblers stick to your last” or “This is our turf”). Insofar even the somehow limited integration of pharmacists in Kinzigtal was far ahead of normal cooperation between pharmacists and physicians (Table 3) [37,38].

**Table 3:**
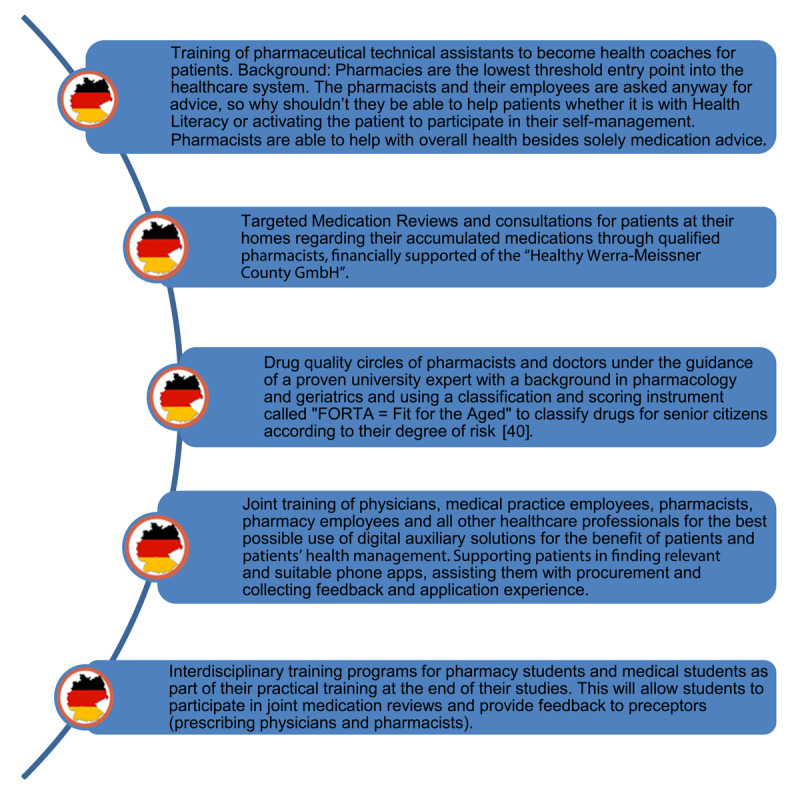
Approaches for partnerships with pharmacists to provide value-based and integrated population health management: © Helmut Hildebrandt, OptiMedis 2019.

### New Federal Law on Remuneration of Pharmaceutical Care

During the last five years a new discussion arose with regard to securing medical care for people mostly in rural areas. Young doctors tend to stay in urban areas and refrain from investing in their own practices i.e., tend to look for salaried positions in medical group practices or so called “medical care centers” owned by hospitals or chains. In rural areas most single-doctor primary care clinics (surgeries) are still solo-practices owned by doctors, which is not the favorite model for young doctors. Insofar some medical practices in rural areas have problems to find replacements and are being shut down, leading to significant access barriers for the rural population. The shortage of physicians and other medical workforce led to new discussions about the division of labor between pharmacists and physicians; therefore, some pharmacists advocated for permission to give flu shots in pharmacies, to consult chronic care patients in disease management, to support medication-use optimization and to repeat chronic care medication prescriptions for a certain period so that the patient does not need to go every month to the medical surgery. A new law from the ministry of health was just processed that proposes experiments in these forms of pharmaceutical care and models for remuneration [Gesetz zur Stärkung der Vor-Ort-Apotheken, 2019]. The professional associations of physicians disagreed but the head of the organization of pharmacists in Germany ABDA made a press release urging for new pharmaceutical care to be remunerated and detailing the possible interventions [37,38,39].

### Pharmacists in the Forefront of a New Population Health Management Pilot “Gesunder Werra-Meissner Kreis”

OptiMedis started in January 2019 a third PHM region in the “Werra-Meissner Kreis [County]” in northern Hesse, following the Kinzigtal model, and looked for a closer partnership with pharmacies from the beginning. Table 3 highlights the approaches for partnering with pharmacies.

Currently, the “Healthy Werra-Meissner County GmbH” is in the process of developing a remuneration model (alternative payment model) for pharmacists that are partnering in these approaches and partnering in population health management activities in this county. A remuneration that is partly activity-based and partly outcome-based. The latter will be the most difficult part of the remuneration model.

At the same time, OptiMedis is working on advancing the **polypharmacy classification instrument** “FORTA = Fit for the Aged” into an artificial intelligence instrument that will be able to assist physicians and pharmacists in using the best appropriate medication for multiple morbidities in elderly patients. In Werra-Meissner, doctors and pharmacists will then be asked and electronically supported to use this software tool [40].

### A Positive Outlook for the Future

In the near future, some of the above mentioned approaches could possibly be made available Germany-wide; if the legislative process that is currently underway succeeds over the resistance of the medical associations and can financially support and facilitate such solutions in the area of “pharmaceutical care”.

Currently pharmacists are mainly remunerated by sales volume and are not incentivized to the best health outcome for a population. Therefore, conflict of interest is an element that cannot be ignored throughout this discussion as well, given the history of pharmacists, physicians and pharmaceutical industry’s aligned interests. Moving towards value-based integrated care, being remunerated for pharmaceutical care (and even better partly outcome-based) offers pharmacists new business models that reduce the current incentives towards selling more drugs instead of consulting patients in the best interest of their overall health.

## Conclusions

Pharmacists are among the most trusted professionals in the healthcare industry due to their knowledge about medications, their compassion for the community, their accessibility to patients and the evidence of successful pharmacists-led clinical and non-clinical interventions. The pharmacy profession has moved to a Doctorate level degree in the U.S. and globally is advocating and aligning with the future of value-based care. Pharmacists are now able to accept more responsibilities and in some cases receive proper reimbursements for their interventions.

In the new era of value-based and integrated care, pharmacists may find their niche in population health management by entering strategic partnerships and alternative payment models to manage costs and improve health outcomes for their patient population. In addition, qualified staff in pharmacies may also assist with health coaching activities like mentioned in the German case. Despite the turf war between scopes of practice among all healthcare professions, by keeping patients first and politics last—we can achieve the Triple and Quadruple Aim [6]. Pharmacists’ impact on population health outcomes and healthcare costs in the value-based care era should continue to be assessed in integrated care settings.
